# Protected Areas’ Impacts on Brazilian Amazon Deforestation: Examining Conservation – Development Interactions to Inform Planning

**DOI:** 10.1371/journal.pone.0129460

**Published:** 2015-07-30

**Authors:** Alexander Pfaff, Juan Robalino, Diego Herrera, Catalina Sandoval

**Affiliations:** 1 Duke University, Durham, North Carolina, United States of America; 2 Centro Agronomico Tropical de Investigacion y Ensenanza (CATIE), Turrialba, Costa Rica; 3 Environment for Development initiative (EfD) at the U. of Gothenburg, Gothenburg, Sweden; University of Massachusetts, UNITED STATES

## Abstract

Protected areas are the leading forest conservation policy for species and ecoservices goals and they may feature in climate policy if countries with tropical forest rely on familiar tools. For Brazil's Legal Amazon, we estimate the average impact of protection upon deforestation and show how protected areas’ forest impacts vary significantly with development pressure. We use matching, i.e., comparisons that are apples-to-apples in observed land characteristics, to address the fact that protected areas (PAs) tend to be located on lands facing less pressure. Correcting for that location bias lowers our estimates of PAs’ forest impacts by roughly half. Further, it reveals significant variation in PA impacts along development-related dimensions: for example, the PAs that are closer to roads and the PAs closer to cities have higher impact. Planners have multiple conservation and development goals, and are constrained by cost, yet still conservation planning should reflect what our results imply about future impacts of PAs.

## Introduction

Do protected areas “work”? Billions are spent annually on tens of thousands of protected areas [1,2,3], yet rigorous evaluation of their impact on deforestation is lacking in much of the literature. One sizeable literature (e.g., [4,5,6,7,8,9,10,11,12,13]) considers 'optimal' reserve location by focusing on species presence while ignoring the variation in baseline clearing pressures that determine how much habitat increases due to a well-enforced protected area (PA). We emphasize that, depending on the baseline, pristine forest in PAs may not indicate an impact: if that forest would have remained pristine without any policy, the PA did not make a difference.

Thus, we cannot assume significant forest impact, as impact will vary greatly across a landscape. We show this for one of the most important active conservation frontiers, the Brazilian Amazon.

The importance of understanding the conditions under which PAs have impact is rising. Efforts to mitigate climate change have increased the attention paid to deforestation's emissions while protection motivated by species conservation will continue (e.g., Convention on Biological Diversity’s Work Program on Protected Areas (www.cbd.int/protected/targets.shtml refers to its "2010 targets") suggests significant benefits from the additional expansion of protected areas). Global policies might reward countries for lowering carbon emissions below an agreed baseline, yet any carbon buyer should demand credible evidence that the emissions from forests really fell. Thus, while new investments in protected areas could be induced by growing carbon markets, the outcomes that would result from such investments are likely to be scrutinized relatively closely and a lack of convincing evidence concerning impacts could be grounds to ignore tropical forest as a potential source of emissions reductions. That would lower funding for forest conservation.

Our examination of Brazil’s Amazon builds on prior work on other countries—e.g., for Costa Rica, [14] for average PA impact and [15]—noting also [16,17] on global patterns. Looking ahead, the Amazon is the world's largest remaining forest frontier. The fate of most Amazon forest remains to be determined, with considerable efforts already expended on development and conservation. Brazil's Amazon has hosted the establishment of extensive protected areas and indigenous lands, including in particular during time periods (late 1990s, early 2000s) that preceeded the periods of deforestation that we study here (2000–2004, 2004–2008). Thus, it is an important case to study.

We extend a limited prior literature on PAs' impacts across the whole Brazilian Amazon (many people study local Amazon conservation, e.g., [18,19]). A leading analysis is [20] concerning impacts on 1997–2000 and 2000–2008 deforestation, with different methods and outputs from ours. Their focus is the average impact across PA types. Results indicate inhibition of deforestation by three of four major PA types (while [21] add a comment that for a given level of baseline deforestation pressure, which we show determines impacts, the PAs with stricter enforcement generate greater impacts). Our focus is the variation across space in PAs' impacts, based on the variation across a landscape in the baseline deforestation pressure that PAs block. We employ propensity-score matching (noted but not used in [20]) to transparently document a role within impact of *where PAs are located within the Amazonian frontier*. We show that it is critical to correct for PAs' bias towards lower pressure. Also, by splitting our samples according to levels of pressure, we show that conservation could be targeted with an explicit goal of increasing impacts of PAs.

Another analytic choice reflects insights from [22], which analyzes various actions other than protection taken to lower deforestation rates in the Brazilian Amazon (e.g., shifts in forest code, rise in enforcement, creation of a federal blacklist and local reactions). That some actions start in 2004, a true breakpoint in the sequence of Amazon deforestation rates, explains why we broke 2000–2008 deforestation up into two periods, 2000–2004 and 2004–2008. Further, as a shift in regional 'deforestation regime' could include a shift in 'conservation regime', we consider the PAs created during 2000–2004 separately from the PAs created before 2000.

To infer the PA impacts of interest, we need to use forest outcomes for unprotected areas to estimate a PA baseline, i.e., what would have happened to protected forests if not protected. Many prior baselines have been based upon deforestation in all unprotected land or next to PAs (see, e.g., [23,24] and literature review in [25]), which fails to produce similar control or comparison observations if PAs' locations are biased (see, e.g., [26,27,28] and, globally, [16] is concerned with how PA sites are delineated but also possible leakage). PAs could be on high-pressure lands if planners target highest impact [29]. They could be on lower pressure lands if planners endeavored to avoid any high cost (financial or political) of establishing PAs. Land prices and protests over PAs may rise with parcel profits. Globally on average, albeit with exceptions, PAs go to low pressure land [16].

To explicitly highlight and address this issue, we employ matching methods to estimate average PA deforestation impact, during both 2000–2004 and 2004–2008, as well as how impact reliably differs in ways we should expect across the PAs located in different parts of the frontier. We find that in both periods PAs do significantly lower the deforestation within their boundaries, albeit less in the second time period when the influences of other policies already lower clearing. Further, we show that when estimating impact it is important to search for similar control points. Since PAs are clearly biased towards lands that are facing lower clearing threat, as we document, our matching estimates of PAs' impacts are, roughly, only half as large as the simplest estimates. Averaging across periods, we find a ~2% reduction in deforestation within the PA' boundaries, while the simplest estimation approach ignoring land characteristics would suggest roughly ~4%.

Yet to inform policy choice we want to go beyond average impact to *variation in impact* as often conservation planners may wish to respond to knowledge of where PA impact is higher. However one counts costs and benefits, that the reduction in deforestation rates in expectation should be at least twice as high for some potential PA locations could be determinative in siting.

Thus next we break the protected areas up into subsets using dimensions of the landscape which are likely to matter to rates of deforestation: distance to the nearest road; distance to a city; rainfall; soil fertility; and slope—all of those factors having been shown to matter to PA impact. For each dimension, we estimate impact for the subsets of PAs we suspect face higher pressure, e.g., closer to the nearest road, as well as the subsets of PAs that we suspect face lower pressure, e.g., farther from the nearest road. Thus, we split our sample using expected levels of pressure and these breakdowns show that the impact of protection varies critically across the landscape. For instance, using controls for matching just as we did in estimating the average impact of PAs, we find the impact of PAs closer to roads to be almost double that of the PAs farther from roads. Further, the impact of the PAs closer to cities is over triple that of the PAs farther from any city. Along the same lines but using a naturally occurring feature of the landscape, impact on 2004–08 deforestation is over twice as high for PAs on high fertility soils versus on lower fertility soils. Finally, a clear indicator of spatially varying deforestation regime is a split into east versus west. That summarizes differences such as just discussed to show much higher PA impacts in the east.

The rest of the paper proceeds as follows. Section 2 has background on land-use choice and evaluations of protection’s impact on land use and land cover. Section 3 describes the data and our matching approach. Section 4 then presents our results and finally Section 5 concludes.

## Protection's Impacts: Theory and Literature

### 2.1 Land Use's Implications For PA Impact

**Fig 1:**
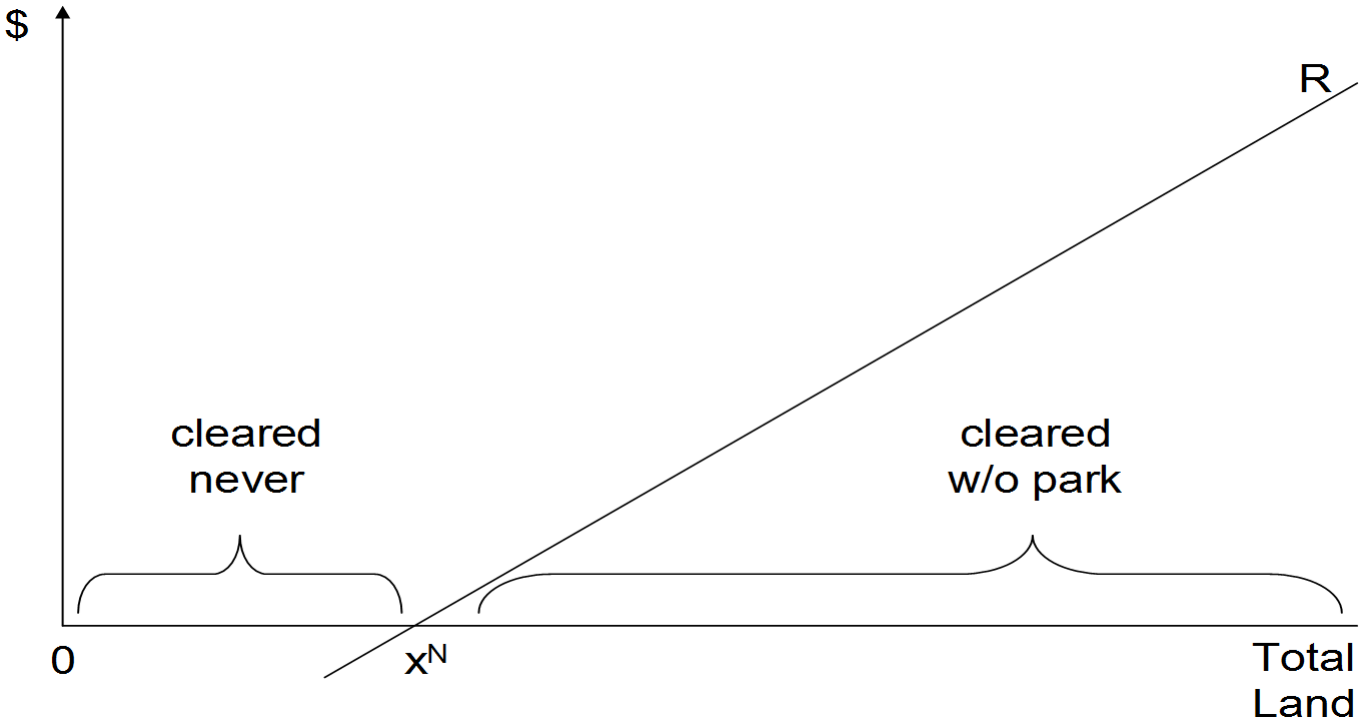
Profitability & Private Land-Use Choice.

Fig 1 presents a simple but useful framework for considering protected areas’ forest impacts. Profitability in production such as agriculture creates opportunity costs of keeping land in forest. In Fig 1, forest land is ordered according to those private rents, with profit rising to the right. Where net private rents are greater than zero, land will be deforested in the absence of protection. However, where net private rents from clearing and producing are negative, land stays in forest even without a PA. Lacking PAs, then, deforestation will take place only above x^N^ in Fig 1.

Putting that another way, protection can lower clearing only within the interval above x^N^. Thus, a protected area’s forest impact depends upon the fraction of its land that is in that interval. If that 'threatened fraction' equals 1, every parcel protected represents avoidance of deforestation. We will estimate what fraction of protected lands would have been deforested, if not protected, using deforestation rates on the unprotected locations most similar to parcels in protected areas. If a large fraction of the similar land was deforested, we will estimate larger impacts from PAs.

Considering some estimation challenges that are clear here, plus considered by matching, we note that if all of the lands and only the lands that are above x^N^ have been placed within PAs, then it is impossible to find unprotected locations just like protected sites other than in PA status. In that case, we would underestimate PA impact at zero by examining the unprotected locations. The same would be true if all of the land and only the land below x^N^ has been placed in PAs, although in that case we would overestimate PA impact at one by examining the unprotected. Thus, Fig 1 makes quite clear that the accuracy of the impact estimate depends on matching, i.e., one way or another comparing protected parcels with unprotected parcels that are similar.

### 2.2 PA Impact Evaluations (without and with land characteristics)

[25] review the protected-area-evaluation literature (noting other reviews in [30,31,32]). They emphasize hurdles for solid inferences about protection’s forest impacts—in light of their earlier documentation [16] that globally, on average, the distribution of PAs over countries’ landscape is not 'as if random' but, instead, biased in deforestation-relevant ways.

Protected areas’ impacts have been evaluated but the methods for doing so have varied. At least some informal evaluations would not appear to involve comparisons at all and instead only observations, e.g. common statements that Costa Rica’s protected areas are a success since they are essentially fully forested. In this vein but the other direction, [33] suggest that PAs are not viable in Kalimantan given considerable deforestation during 1996–2002. Either sort of conclusion based solely on current forest can go wrong since true impact is a difference between current forest and what would have happened in a protected area without the protection. While not including such a baseline is understandable, given that it cannot actually be observed, still it is worth trying to estimate one empirically. Several options for doing so have been tried.

For instance, some past evaluations compare outcomes in PAs' boundaries to outcomes in all unprotected areas. [34] discusses deforestation rates for all unprotected areas from 1972 to 2002, comparing a ~2.9% annual unprotected clearing rate with lower rates in PAs. One is tempted to conclude from such comparisons that the protection lowered deforestation and other analyses and claims along these lines are [35] on the Ecuadorian Amazon, [36] for Sarapiqui in Costa Rica and, globally, [37].

More commonly, past evaluations have compared PAs with the outcomes in nearby areas. [23] considered deforestation in and around 93 PAs in 22 tropical countries using survey data. [38] noted deforestation was equal to or higher inside the reserve than in a 3km buffer zone (extended later by Viña et al. 2007). [36] analyzed 1960–1997 deforestation in and around 132 PAs using 0.5, 1.0, and 10.0km contiguous zones. [39] compare a northern Guatemalan Maya Biosphere reserve to surrounding land for four time periods. [40] provide a detailed study of Gunung Palung National Park (GPNP) comparing to a 10km zone around the park. Generally, comparisons making use of this approach find lower deforestation rates within PAs and thereby claim evidence of impacts.

However, PAs may be less deforested due to protection or because the characteristics of their lands do not promote deforestation. If so, protection itself could have little or zero impact. The literatures above assumed that either all unprotected lands or nearby ones are similar to PAs. While either could be true in any given case, matching documents explicitly a difference in land characteristics, if one exists, then searches explicitly for lands with similar such characteristics.

That matching method—which is the method we apply here—has been demonstrated for a leading country in developing of PA networks, Costa Rica. For 1960–1997 deforestation, [14] use matching to similar unprotected for points in over 150 PAs to control for land productivity and distances from forest edge, roads, and cities. Matching greatly increased each covariate’s similarity and yielded an estimate that approximately 11% of PA points would have been deforested without protection (this result was robust to requiring very high similarity). That stands in striking contrast to the impact estimate of 44% if comparing to all unprotected. Even comparing to nearby unprotected, which in principle can help a lot, estimated 38% impact. [17] demonstrate that such results from matching's process—for addressing the bias in PAs' locations towards areas that face lower clearing pressure—are globally relevant. For global data (meaning less precise data and fewer controls), like [14] they find that average ‘apples-to-apples' estimates are under half of estimates from using all unprotected.

Yet guiding policy may require going beyond average impacts to highlighting variation in impacts, so that policies could target circumstances with higher impacts. Thus, [15] revisit Costa Rican PAs using matching for 1986–1997 and found significant variation in impact. PAs within 85 km of San Jose, a major metropolitan area, avoided 3% deforestation while those further from San Jose avoided only about 1%. PAs within 6 km of a national road are estimated to have avoided 5% deforestation while in forests farther away, PA impact was essentially zero. Slope, which in Costa Rica is a good proxy for agricultural suitability, was a critical factor. PAs on flatter land blocked 14% deforestation but the PAs on steeper lands had close to zero impact.

However, while Costa Rica has been a policy leader, application of these kinds of lessons and, more generally, methods will not have its greatest impacts in the future within Costa Rica. The Brazilian Amazon is a much larger forest area (the Legal Amazon is ~100 times the size of Costa Rica) and a more active deforestation frontier. Good evaluation for the Amazon is critical.

Along these lines, we note that in addition to work discussed above, [41] and [42] provide more local application of matching to PAs in Brazilian Amazonia. Both highlight the key role of the baseline in showing that ‘apples to apples’ comparisons can be supportive of estimated PA impacts when PAs are in areas with relatively high threat of clearing. Both concern the state of Acre. Starting more locally, [41] consider the Chico Mendes extractive reserve which has incurred non-trivial deforestation (some extraction is legal). However, it is close to the InterOceanic Highway—much more so than is the average for Acre—and comparing deforestation in Mendes to other places under high pressure shows a PA impact. [42] build upon that to compare all 'sustainable use' PAs in Acre, which allow some clearing legally, with more strict 'integral' areas. The latter are pristine but because their sites do not face much clearing pressure, the former were estimated to have avoided more deforestation.

## Data & Empirical Strategy

### 3.1 Dependent & Independent Variables

#### 3.1.1 Deforestation

We study 2000–2004 and 2004–2008 deforestation in Acre using PRODES data on land cover for 2000, 2004 and 2008 from INPE (*Instituto Nacional de Pesquisas Espaciais*). For a single pixel, the data indicate one land-cover class. Thus, deforestation is a change from forest to a non-forest land cover. For each forest pixel in 2000, our deforestation variable is binary (value = 1 for forest in 2000 but not in 2004, and value = 0 for forest in both years) and for each forest pixel in 2004, again deforestation is binary (value 1 if forest in 2004 but not 2008, and 0 if forest in both years).

The original PRODES dataset was downloaded in raster format from INPE’s website in Geographic Coordinate System, South American Datum of 1969. The cell resolution of the raster was 0.000808 decimal degrees, which is equivalent to 2.9088 s, or 90 m around the equator once projected. INPE’s own analyses, since the year 2001, in fact are conducted at a finer scale, then the results are resampled to 90x90 m in order to create the down-loadable version of these data.

#### 3.1.2 Protected Areas

The Brazilian Legal Amazon is a region of 521,742,300 hectares (approximately 5 million km^2^). About 30 percent of the Legal Amazon is under one of the following forms of protection, within one of 532 total protected areas. We consider them on aggregate and as East (Para, Amapa, Mato Grosso, Tocantins, Maranhao) versus West (Acre, Rondonia, Amazonas, Roraima), as well as in subsets defined by relatively high or low distances to roads, cities and forest edge (more below).

**Fig 2:**
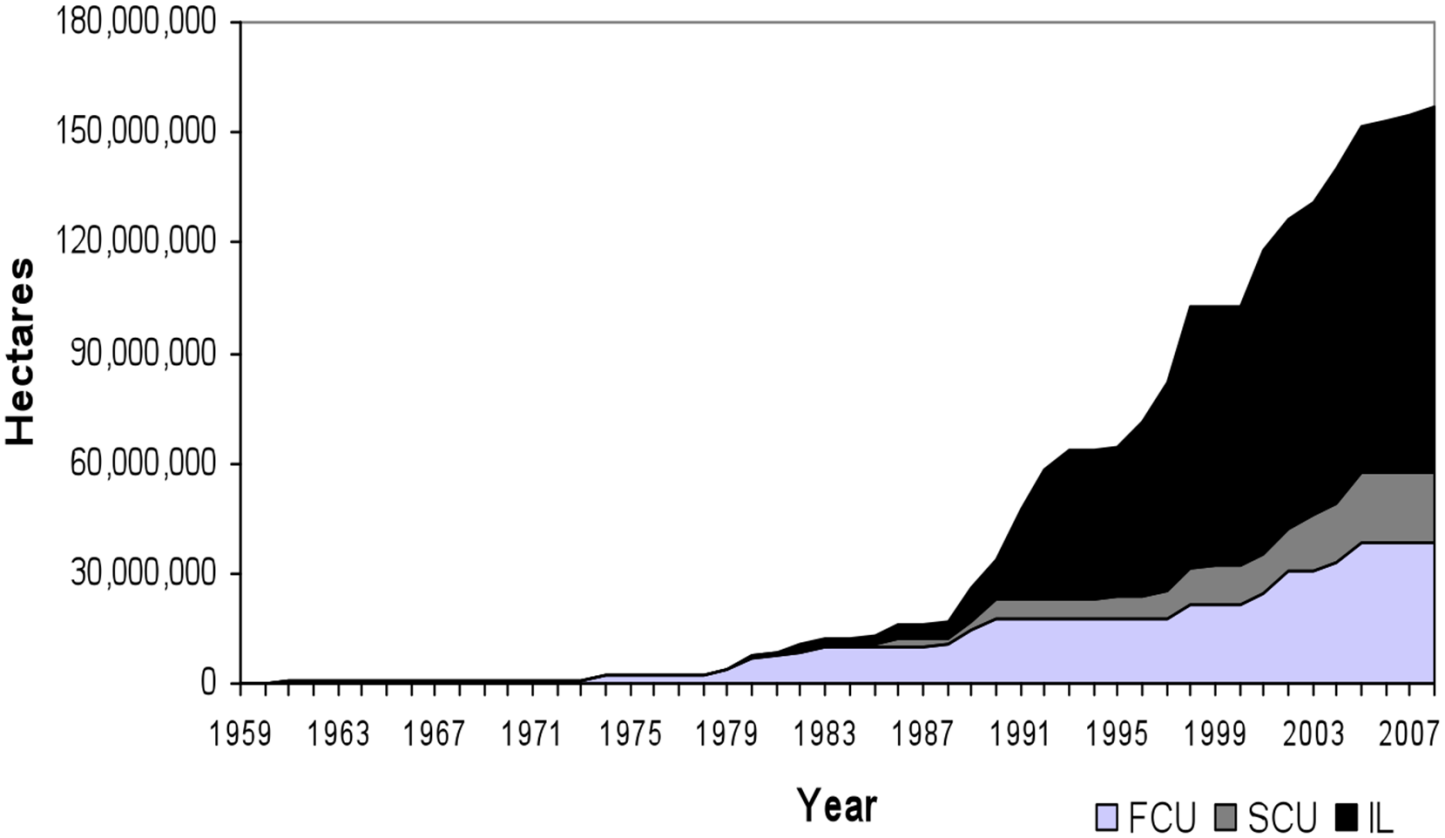
Creation of Protected Areas: hectares by year (cumulative).

Fig 2 presents the number of protected hectares for these categories during 1959–2008. Most of protected areas were implemented during 1990–1999, especially in the cases of the State Conservation Units and the Indigenous Lands. However, most of the land that has been protected within Federal Conservation Units was protected during the 1980–1989 and 2000–2008 periods. Again considering this on the whole, most of the PAs were created before our 2000–2004 period of deforestation, while of the rest a large share were created before our 2004–2008 time period.

Within our analyses, a point is treated as 'protected' if the PA it is in was created before the deforestation being analyzed. Pixels in PAs created after 2008 are controls, as they were not in PAs during our deforestation periods. Pixels in PAs created during any period of deforestation are not included within those analyses, as we cannot tell if deforestation proceeded them or not.

#### 3.1.3 Land & Site Characteristics

Many factors that affect the benefits, the direct costs and the opportunity costs of clearing forests have influence upon deforestation decisions. For the same reasons, since the relative profitability of land could lead local stakeholders to resist the establishment of protection—as it restricts their productive activities—many of those same factors can be expected to influence the siting of PAs. Factors that affect both types of decisions are liable to bias empirical estimates of PAs' impacts.

Thus, to correctly infer the impacts of protected areas on deforestation rates, we need to control for the influences of the factors we observe that affect the profitability of deforestation. That includes relevant characteristics of a location that are not physical features of the land itself, such as the distance to the nearest road in 1985 (date chosen because it is before most protection) as well as the distances to the nearest city in 1991 (date chosen again to come before protection). Another relevant distance is that to the forest’s edge. For analyses of 2000–04 deforestation, we use distance to forest edge in 2000, while for 2004–08 deforestation, we use that distance in 2004—in both cases employing the same datasets for deforestation that we have just discussed above. Digital road maps were provided by the Department of Geography at Michigan State University, based on paper maps by DNER (*Departamento Nacional de Estradas de Rodagem*), an agency in the Transport Ministry in Brazil, while 1991 cities information is from the Demographic Census.

We also use maps of relevant biophysical conditions. We employ an index of soil quality, rainfall from [43], vegetation type (cerrado/not) and a binary indicator of slope (distinguishing, e.g., “steeply sloped” from “rolling hills”) from the 'Diagnostico' data of IBGE (*Instituto Brasileiro de Geografia e Estatistica*). Table 1 confirms the relevance of such factors concerning deforestation, while Table 2's descriptive statistics suggest they affect PA siting too.

**Table 1:** Deforestation On Unprotected Land.

	Deforestation 2000–04	Deforestation 2004–08
log (Road Distance)	-0.0113***	-0.0033***
	[0.000]	[0.000]
log (City Distance)	-0.0405***	-0.0093***
	[0.001]	[0.001]
log (Edge Distance)	-0.0022***	-0.0182***
	[0.000]	[0.000]
Flatter Slopes	0.0031***	0.0040***
	[0.001]	[0.001]
Soil Fertility	-0.0049***	0.0008**
	[0.000]	[0.000]
Rainfall Amount	-0.0000**	-0.0000***
	[0.000]	[0.000]
Vegetation Type	-0.0427***	-0.0050***
	[0.002]	[0.002]
Rondonia & Acre	0.0261***	0.0318***
	[0.002]	[0.002]
Maranhao & Tocantins	0.4230***	0.0742***
	[0.003]	[0.003]
Paraná & Amapa	0.0541***	0.0209***
	[0.001]	[0.001]
Mato Grosso	0.0688***	0.0563***
	[0.001]	[0.001]
constant	0.6223***	0.3285***
	[0.007]	[0.006]
# obs	328,810	279,473

**Table 2:** Descriptive Statistics.

In 2000	# obs.	2000–2004 Deforestation (%)	Road Distance (m)	City Distance (m)	Edge Distance (m)	Rainfall Amount (mm)	Vegetation Type (1/0)	Soil Fertility (1–5 = best)	Flatter Slopes (%)	Rondonia Acre (%)	Maranhao Tocantins (%)	Paraná Amapa (%)	Mato Grosso (%)	Amazonia Roraima (%)
**Non Protected Areas**	328,810	5.52	90,142	80,591	3,478	2,300	0.04	3.03	0.60	6.94	2.81	32.25	13.01	44.99
**Protected Areas before 2000**	124,283	0.61	114,629	114,339	4,023	2,333	0.04	2.99	0.57	14.85	1.64	29.80	7.23	46.46
In 2004	# obs.	2004–2008 Deforestation (%)	Road Distance (m)	City Distance (m)	Edge Distance (m)	Rainfall Amount (mm)	Vegetation Type (1/0)	Soil Fertility (1–5 = best)	Flatter Slopes (%)	Rondonia Acre (%)	Maranhao Tocantins (%)	Paraná Amapa (%)	Mato Grosso (%)	Amazonia Roraima (%)
**Non Protected Areas**	279,473	3.82	90,470	79,673	2,989	2,312	0.05	3.06	0.58	6.92	1.51	31.04	13.26	47.27
**Protected Areas before 2000**	122,578	0.46	114,881	115,118	3,753	2,334	0.04	2.99	0.57	14.83	1.49	30.10	7.13	46.44
**Protected Areas 2000–2004**	36,285	0.07	128,343	107,946	4,567	2,489	0.02	3.20	0.34	7.16	0.00	17.01	12.73	63.10

#### 3.1.4 Observations & Our Sample

We start with a sample of 800,000 pixels. If the land-cover information available (16 categories) does not clearly indicate that the point was in forest cover, then we simply drop the observation (including the categories *No Data*, *Non Forest*, *Water*, *Clouds*, and *Residual*), leaving us with a sample of ~450,000 pixels in forest in 2000 (and in 2004) that can be examined for deforestation.

### 3.2 Matching Approach

If protection in the Brazilian Amazon had been implemented randomly across all forest lands, its impact on deforestation would be easy to estimate. We would only need to look at the difference between the deforestation rate inside and outside of the protected areas. The deforestation rate outside would be an unbiased estimate of what would have been the deforestation rate inside the boundaries of protection had there been no protection since the other factors would cancel out.

However, PAs do not appear to have been located in a 'random-like' fashion. Of course, we know they were not actually randomly sited, in the sense of flipping coins or throwing darts, but the key question is whether there is any bias along dimensions that affect deforestation rate. Table 2 shows that relevant land and site characteristics—including distances to road and city—of the lands within the PAs differ from those same characteristics for all of the unprotected lands.

Both basic land-use theory and past work suggest that such differences will affect forest. Thus the observed differences in deforestation rates between protected and unprotected reflect not only any impacts of protection but also the influences of these differences in characteristics. To remove those influences, we use matching techniques. The principle of this technique is to find an improved and acceptable control group by matching each treated observation to the most similar untreated observations, for more of an ‘apples to apples’ comparison. Thus the land under protection is compared not to all unprotected land but only the most similar unprotected subset.

To define ‘similarity’, in applying propensity-score matching we use the probability of a pixel being protected. Thus, protected-pixel outcomes are compared to deforestation in pixels not protected but with similar enough characteristics to yield a similar probability of being protected. The probabilities used to do this are generate by a probit model for being protected, i.e. for being in a park or not, with regressors being all covariates of treatment [44].

With similarity thus defined, we choose how many untreated observations to compare to each treated observation. There is a tradeoff. As the number of matches increase, the variance of the estimator will decrease because it will be based on more data. However, the bias will increase because we have gone beyond the most similar unprotected pixel to ever more dissimilar pixels. We also check robustness dropping controls nearby (20km), in case PAs have nearby spillovers.

Given an adequate control group, we estimate the counterfactual deforestation for the protected area (had it not been protected) and compare that with the actual park deforestation. We also run a regression using the treated and the matched untreated parcels, with the treatment dummy included to get the estimated effect of protection controlling for any residual differences between treated and untreated in terms of all of the other relevant factors that we have observed. This helps to minimize biases generated by other variables that affect deforestation decisions.

## Results

Table 1 presents a regression examining rates of deforestation for the lands that are not protected, confirming the significance of factors in deforestation for which we will control in testing PAs' impacts on deforestation. The coefficients for the first set of variables confirm that distance from the nearest road, the nearest city and the edge of the forest all lower the chance of a forest pixel being cleared. Among the biophysical measures, for instance, lower slope increases the rates of clearing while having worse soil (for which the fertility index is higher) lowers deforestation. Finally, we can see that the states differ considerably, consistent with distinct regional dynamics.

### 4.1 Identifying & Matching Where Protection Occurred

#### 4.1.1 Protected vs. All Unprotected

Table 2 shows that land characteristics of the unprotected land differ from the protected areas. The outcomes clearly differ as well, providing a reason to understand characteristics differences. Deforestation during 2000–2004, for instance, is on the order of half a percent within the PAs but over five percent in unprotected areas. For 2004–2008, it is almost four percent outside the PAs, while again it is on the order of half a percent within the PAs that were established before 2000. Interestingly, it is under a tenth of a percent within the PAs that were created during 2000–2004. Such deforestation differences lead one to ask whether they represent impact or land differences.

Turning to land and site characteristics, the first such column shows that not only are our sample pixels within the PAs established before 2000 clearly farther from the nearest road than the unprotected pixels, but also the newest PAs created during 2000–2004 are even farther. The second characteristics column shows that the difference for earlier PAs versus unprotected is even greater for the distance to the nearest city, with the newest PAs this time a little bit closer. Concerning the distance to the forest’s edge, again protected pixels are farther than unprotected. All of these differences, which are important in Table 1, i.e., are relevant for deforestation rates, may imply that the differences in deforestation outcomes in PAs are not actually impacts of PAs.

The differences along biophysical dimensions in Table 2’s columns are less pronounced, with very little percent difference between the PAs created before 2000 and unprotected pixels. There is somewhat more difference for new PAs—which again could reflect different thinking—with the rainfall index being a bit higher, vegetation lower and fraction of flat land clearly lower. Finally, recalling they matter in Table 1, there are clear differences in distributions across states.

Table 3 examines Table 2’s facts in a multivariate regression so that each such difference is examined with controls for the other differences in order to see whether on its own it mattered concerning where protection occurred. Probit regressions explain whether a pixel was protected and, in short, for the two siting decisions for protection (i.e., before 2000 and during 2000–2004) the results show the statistical significance of differences we documented simply within Table 2. Summarizing some key examples, all those distances that decreased the probability of clearing also increase the probability of protection being established (for forest edge, only in 2000–2004). That combination suggests a default bias towards an overestimation of the impacts of these PAs.

**Table 3:** Protection's Locations.

	Protected Areas Before 2000	Protected Areas 2000–2004
log (Road Distance)	0.1088***	0.2929***
	[0.002]	[0.004]
log (City Distance)	0.4886***	0.4160***
	[0.003]	[0.006]
log (Edge Distance)	0.0005	0.0199***
	[0.001]	[0.002]
Flatter Slopes	0.0484***	-0.4443***
	[0.004]	[0.007]
Soil Fertility	-0.0302***	0.1092***
	[0.002]	[0.004]
Rainfall Amount	0.0001***	0.0004***
	[0.000]	[0.000]
Vegetation Type	0.0496***	-0.0108
	[0.011]	[0.020]
Rondonia & Acre	0.8978***	0.4146***
	[0.008]	[0.015]
Maranhao & Tocantins	0.5218***	-
	[0.017]	-
Paraná & Amapa	-0.0063	-0.3295***
	[0.006]	[0.010]
Mato Grosso	-0.0079	0.6236***
	[0.008]	[0.012]
constant	-7.4725***	-10.4557***
	[0.043]	[0.081]
# obs	453,093	311,537

#### 4.1.2 Protected vs. Matched Unprotected

**Fig 3:**
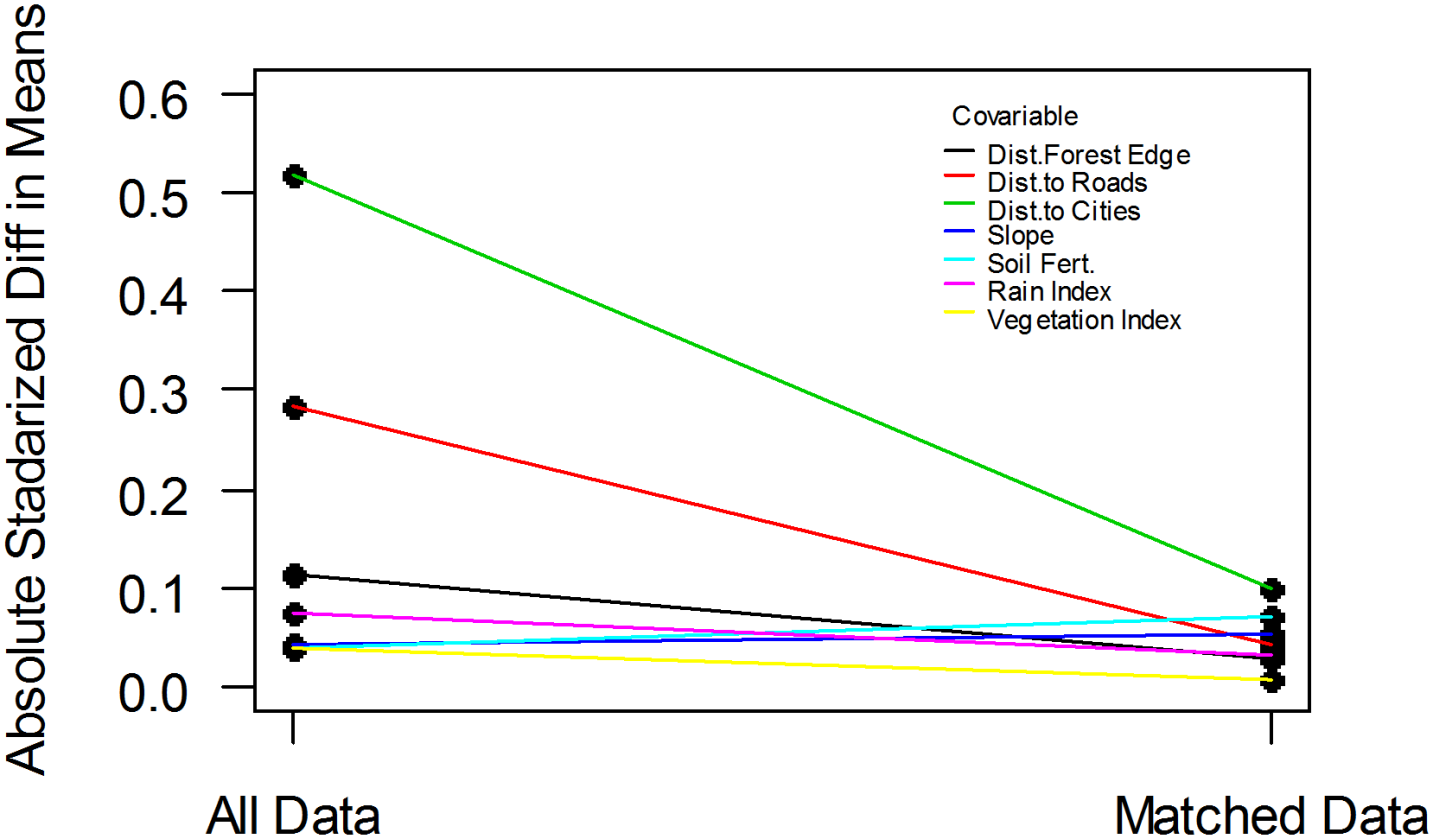
Matching Improves Balance (all, high vs. low road distance, pre-2000 vs. 2000–04).

While protection clearly occurred on non-random land (to what effect we discuss below), there is enough data concerning and enough variation within unprotected pixels to find matches which greatly reduce the differences in land and site characteristics compared to the protected pixels. Fig 3 conveys that matching greatly reduced the differences in land and site characteristics between the treated pixels and the unprotected pixels we selected. Putting that another way, the most similar unprotected pixels were far more similar on average.

Naturally, there are many ways to measure how much more similar or ‘apples to apples’, comparing to protected pixels, were the matched unprotected pixel pools versus all unprotected. To facilitate side by side examination of all of the land and site characteristics that we employed, Fig 3 employs an absolute standardized difference in means to represent each of those factors. The left side of the figure shows the differences between protected and all unprotected pixels and we can see that while some initial differences were relatively small, others were relatively large. For instance, the important distances to city and road started out with large percent differences. However, as conveyed by the slope of their lines, those differences are significantly reduced by the matching. After matching, all of the differences are roughly between zero and ten percent.

Yet two caveats are worth noting. First, looking across Fig 3's subfigures for different matching analyses that we did, we can see that both initial differences and reductions can differ, which would be expected because sometimes we had already focused on distance-based subsets (and it is interesting to see that there is still selection within those, so matching is still helping). Yet generally, city and road distances are among larger initial differences and they get reduced. Second, it is important to say that here we are commenting only on the means of characteristics. At the pixel level, differences will remain. We address those using post-matching regressions.

### 4.2 Estimating PA Impacts: average & predictable variation

Table 4 presents our matching estimates of PA impact alongside other estimates for comparison. To see how controlling for characteristics compares to mean outcomes from Table 2, we include the protected and unprotected means to easily see how much of their difference is due to siting. To explore robustness in controls for observables, we also include standard OLS regressions that can be challenged by severe differences in locations but here provide reassuringly similar results.

**Table 4:** PAs' Deforestation Impacts 2000–2004 & 2004–2008: averages & pressure-level gradients.

	*DEFORESTATION 2000–2004*		*DEFORESTATION 2004–2008*		*DEFORESTATION 2004–2008*	
	All Parks Before 2000		All Parks Before 2000		New Parks 2000–2004	
*ALL*	*All*		*All*		*All*	
*# obs*	*124*,*283*		*122*,*578*		*36*,*285*	
**Differences In Means**	-0.049***		-0.034***		-0.038***	
**OLS Regression (all obs)**	-0.024***		-0.022***		-0.014***	
**PSMatch, n = 1 caliper 1%**	-0.023***		-0.020***		-0.015***	
*Splits by Road Distance*	*High*	*Low*	*High*	*Low*	*High*	*Low*
*# obs*	*74*,*647*	*49*,*636*	*71*,*034*	*51*,*544*	*28*,*155*	*8*,*130*
**Differences In Means**	-0.012***	-0.079***	-0.012***	-0.048***	-0.012***	-0.057***
**OLS Regression (all obs)**	-0.009***	-0.047***	-0.012***	-0.032***	-0.011***	-0.026***
**PSMatch, n = 1 caliper 1%**	-0.008***	-0.043***	-0.010***	-0.027***	-0.011***	-0.028***
*Splits by City Distance*	*High*	*Low*	*High*	*Low*	*High*	*Low*
*# obs*	*79*,*374*	*44*,*894*	*77*,*364*	*45*,*204*	*25*,*153*	*11*,*132*
**Differences In Means**	-0.010***	-0.077***	-0.014***	-0.045***	-0.015***	-0.053***
**OLS Regression (all obs)**	-0.008***	-0.056***	-0.012***	-0.034***	-0.012***	-0.015***
**PSMatch, n = 1 caliper 1%**	-0.008***	-0.048***	-0.012***	-0.028***	-0.011***	-0.021***
*Splits by Edge Distance*	*High*	*Low*	*High*	*Low*	*High*	*Low*
*# obs*	*68*,*761*	*55*,*522*	*68*,*548*	*54*,*030*	*22*,*846*	*13*,*439*
**Differences In Means**	-0.027***	-0.070***	-0.004***	-0.057***	-0.005***	-0.065***
**OLS Regression (all obs)**	-0.017***	-0.031***	-0.004***	-0.035***	-0.004***	-0.033***
**PSMatch, n = 1 caliper 1%**	-0.015***	-0.028***	-0.003***	-0.030***	-0.003***	-0.030***
*Splits by Subregion*	*West*	*East*	*West*	*East*	*West*	*East*
*# obs*	*76*,*212*	*48*,*071*	*75*,*110*	*47*,*468*	*25*,*494*	*10*,*791*
**Differences In Means**	-0.0091***	-0.0916***	-0.0078***	-0.0646***	-0.0133***	-0.0657***
**OLS Regression (all obs)**	-0.0050***	-0.0434***	-0.0038***	-0.0392***	-0.0006	-0.0389***
**PSMatch, n = 1 caliper 1%**	-0.0038***	-0.0419***	-0.0027***	-0.0354***	-0.0030***	-0.0379***

#### 4.2.1 Average Impact

Table 4's top row—i.e., the three rows for means, OLS and matching that are in the same box—considers all protected areas at once, with each column providing a different impact evaluation: the 1^st^ column is impact on 2000–2004 deforestation, while the others are for 2004–2008 clearing, the 2^nd^ column being for the PAs created before 2000 and the final column for 2000–2004 PAs. Across columns, the matching (and OLS) impacts are about half as large as those from Table 2. Thus, roughly half of the apparent impact from comparing means is actually due to PA locations, strongly confirming the importance of controls for observed characteristics in testing for impact (and we note that these results are extremely robust to dropping nearby controls pre-matching).

Put another way, these results show that each set of PAs, on average, does have impacts. Concerning 2000–2004 deforestation, it appears a bit more than 2% clearing was avoided within the PAs created before 2000. Those PAs avoided less clearing in the next time period, blocking just under 2% deforestation in 2004–2008, as there was less deforestation. For that latter period, the deforestation impact estimate for the newer PAs created during 2000–2004 is slightly lower, even though the means difference was slightly larger as less clearing occurred within those PAs. That is consistent with those PAs being even further from the nearest road and on less flat lands and we see that separating out 'conservation regimes' over time has value in clarifying impacts.

That said, we must emphasize that matching (and OLS) control only for the observed characteristics. Undoubtedly there can be characteristics we do not observe that could influence both the probabilities of deforestation and the likelihood that protection is established on a site. For a sense of how strong the influences of such unobserved factors would have to be in order to eliminate the conclusions suggested by our matching results, we also calculate the Rosenbaum bounds on matching results. For these central results, for instance, the smallest Gamma is 6, which implies considerable robustness in the conclusions from our controls for observables.

#### 4.2.2 Predictably Varying Impact Across The Landscape

The rest of Table 4 demonstrates that PA impact varies predictably across the landscape—a fact that is as important as the average in considering the establishment of any additional PAs. Table 1 has shown that deforestation pressure varies considerably—and at least to some extent understandably—across space. That is critical for impact because for well-enforced protection—which to first order must be considered the norm in the Brazilian Amazon (not every country)—any PA's deforestation impact equals exactly the deforestation pressure that the PA has blocked. Here, using various ways of breaking up the landscape, we demonstrate this for the Amazon. To not repeat this point for each subset, we note that the differences in impact hold in each column, i.e., variable impact holds for pre-2000 and 2000–2004 PAs and for both deforestation periods.

The second 'meta-row' in Table 4 again shows matching estimates of impact alongside other estimates but now for PA subsets with higher and lower distances to the nearest road (in column 1 the cutoff is 71km while in columns 2 and 3 it is 75km). These subsets very clearly differ in impact: PAs closer to roads avoid about 5 (3) times as much earlier (later) deforestation. While other factors will also influence PA policies, such differences in impacts clearly should.

Table 4's third 'meta-row' provides matching and other estimates of PA forest impact for PA subsets with higher and lower distances to the nearest city (in column 1 the cutoff is 76km while in columns 2 and 3 it is 78km). Once again, these higher and lower pressure subsets clearly differ in impact: PAs closer to cities avoid about 5 (2) times as much earlier (later) deforestation. Thus, easily observable features of development—roads and cities—affect conservation impact.

The penultimate 'meta-row' in Table 4 then utilizes the distance to the forest edge to split the PAs into subsets (in each column the cutoff is about 2km). The reason is that it is difficult to penetrate the forest and thus points far from the edge are less likely to be cleared (as in Table 1). The higher and lower pressure PA subsets again clearly differ in impact: PAs closer to the edge avoid about 2 (10) times as much earlier (later) deforestation. This too can guide conservation.

Finally, the last set of estimates employs our division of the region into East versus West (again, East = Para, Amapa, Mato Grosso, Tocantins, Maranhao while West = Acre, Rondonia, Amazonas, Roraima) that reveals high-pressure (East) impacts always at least ten times as large. That's a dramatic indication of important regional differences relevant for further development and conservation investments as well as their interactions, which can inform integrated planning. In sum, across conservation and deforestation regimes, PAs' impacts clearly rise with pressure.

## 5. Discussion

We extended a limited prior literature on PA impact across the whole Brazilian Amazon by focusing on predictable variation across space in deforestation pressure and, thus, in impacts. On average, PAs lowered 2000–2004 and 2004–2008 deforestation rates within their boundaries (less in the second time period, when the influences of other policies lowered deforestation too), though only half as much as suggested by simpler estimates without controls for PAs' locations.

At least as important for guiding planning are the conservation-development interactions we document by splitting up our PA sample and estimating impact at different levels of pressure. As is predicted by standard, static land-use economics applied to forest clearing of a landscape, the PAs nearest to roads and nearest to cities have considerably higher impacts on deforestation and this point is effectively summarized by much higher deforestation impacts of PAs to the east. Thus, we confirmed that conservation could be targeted with the goal of increasing PA impacts.

This type of result certainly could motivate the exploration of other variations in impacts. Some authors have considered impacts by PA types and that limited literature could be extended. As [45] note, this raises political economy questions on varied dimensions, including potentially the influence of both local stakeholders and the agencies making decisions.

Yet we must concede that larger scale averages mask significant variations even when, as in this paper, authors make some effort to break larger sets of PAs into smaller but still large sets. Putting that another way, local contexts that vary on dimensions both observed and unobserved surely can influence the effects that PAs have upon local behaviors and, thus, upon deforestation. For instance, surely it behooves policy makers to also consider the types of land uses occurring. Further, dynamics of development matter, e.g., marginal impacts of infrastructure differ with prior investments (roads in [46]), affecting optimal integrated conservation planning.

Such dynamics also suggest variation in PA spillovers (versus impacts in PA boundaries). For instance, when a PA is established, individuals producing there may raise production nearby. Alternatively, reduced supplies from protected lands may raise prices and production elsewhere. The details of those spatial response dynamics undoubtedly can vary considerably across settings ([45] note that dynamics differences could imply different spillovers *signs*).

Finally, we must acknowledge that impacts on deforestation rates clearly will not be the only determinants of conservation planning. However, we believe that the implications of results such as those provided here can be blended with foci upon both costs and variations in benefits.
